# A Novel Cobalt Metallopolymer with Redox-Matched Conjugated Organic Backbone via Electropolymerization of a Readily Available N_4_ Cobalt Complex

**DOI:** 10.3390/polym13101667

**Published:** 2021-05-20

**Authors:** Mikhail Karushev

**Affiliations:** Ioffe Physical-Technical Institute of the Russian Academy of Sciences (Ioffe Institute), 26 Politekhnicheskaya str., 194021 St. Petersburg, Russia; mkarushev@mail.ioffe.ru

**Keywords:** conducting metallopolymer, polymeric cobalt(II) complex, electrochemical polymerization, UV-vis-NIR spectroelectrochemistry

## Abstract

Fast and reversible cobalt-centered redox reactions in metallopolymers are the key to using these materials in energy storage, electrocatalytic, and sensing applications. Metal-centered electrochemical activity can be enhanced via redox matching of the conjugated organic backbone and cobalt centers. In this study, we present a novel approach to redox matching via modification of the cobalt coordination site: a conductive electrochemically active polymer was electro-synthesized from [Co(Amben)] complex (Amben = *N,N′*-bis(*o*-aminobenzylidene)ethylenediamine) for the first time. The poly-[Co(Amben)] films were investigated by cyclic voltammetry, electrochemical quartz crystal microbalance (EQCM), in situ UV-vis-NIR spectroelectrochemistry, and in situ conductance measurements between −0.9 and 1.3 V vs. Ag/Ag^+^. The polymer displayed multistep redox processes involving reversible transfer of the total of 1.25 electrons per repeat unit. The findings indicate consecutive formation of three redox states during reversible electrochemical oxidation of the polymer film, which were identified as benzidine radical cations, Co(III) ions, and benzidine di-cations. The Co(II)/Co(III) redox switching is retained in the thick polymer films because it occurs at potentials of high polymer conductivity due to the optimum redox matching of the Co(II)/Co(III) redox pair with the organic conjugated backbone. It makes poly-[Co(Amben)] suitable for various practical applications based on cobalt-mediated redox reactions.

## 1. Introduction

Conductive polymers are of utmost importance in a variety of different scientific and technological applications. In particular, electrochemical modulation of conductive, optical, magnetic, and mechanical properties of conductive polymers is a favorable option to create smart materials [1]. Introduction of a transition metal in the conjugated system significantly affects functional properties of conjugated polymers [2], especially if the metal center exhibits redox activity.

Polymers of Salen-type transition metal complexes represent an interesting class of conjugated metallopolymers. Salen-type complexes are metal complexes with redox active N_2_O_2_ Schiff base ligands that are able to coordinate a variety of metal cations. The term “Salen” refers to the specific ligand *N,N′*-bis(salicylidene)ethylenediamine, prepared via condensation of two equivalents of salicylaldehyde and one equivalent of ethylenediamine. “Salen-type” is commonly used to name structurally similar ligands with various phenyl ring substituents and diamine backbones. The locus of oxidation and the degree of localization/delocalization of the resulting electron hole in Salen-type complexes depends of the metal center, ligand substituents, availability of exogenous ligands, temperature, etc. [3]. Corresponding metallopolymers are readily accessible by oxidation (usually electrochemical) of unprotected Salen-type complexes. The resulting metallopolymers are electrochemically active, electrochromic, conductive in the doped state, and prone to multi-electron redox reactions, which allows their use in energy storage systems [4], electrochromic devices [5], electrocatalytic systems [6], and photoelectrochemical cells [7]. Among various known polymeric Salen-type complexes, Cobalt Salen-type polymers are of special interest because they exhibit both metal- and ligand-based redox activity [8,9,10,11]. This contrasts with more well-studied Ni- and Cu-based Salen-type polymers that are converted from neutral to oxidized state via ligand-based redox reactions involving consecutive formation of cation radicals and di-cations in biphenyl fragments, while metal centers act as structuring and redox-mediating units [12,13,14]. Efficient metal-centered redox reactions in Cobalt Salen-type metallopolymers enhance their capacitive properties [9,15] and make them useful in electrocatalytic [11] and sensing applications [16]. Unfortunately, the Co(II)/Co(III) redox process is usually observed only in very thin films of polymerized [Co(Salen)]-type complexes and diminishes in thick films, which complicates the practical use of these materials. The decreased electroactivity of the cobalt centers in thick polymer films has been previously related to the low conductivity of the conjugated backbone at potentials of the metal-based redox process. Tuning the redox potential of the organic polymer backbone to be the same or below that of the Co(II)/Co(III) redox potential has been proposed as a powerful tool to enhance metal-centered electrochemical activity in conjugated metallopolymers [17,18]. For [Co(Salen)]-type polymers, the introduction of 3,4-Ethylenedioxythiophene (EDOT) or 2,2′-bithiophene units in the *para*-positions of the phenyl rings of the ligand of the starting monomer proved to be an effective strategy for matching redox potentials of the cobalt centers and the organic backbone, but involved the use of complicated synthetic procedures [10,11,19,20]. The potentials of metal- and ligand-based oxidations of metallocomplexes have also been shown to depend on the type of heteroatoms in the metal coordination site [3]. The modification of the N_2_O_2_ coordination sphere of [Co(Salen)]-type complexes could therefore potentially be a simple approach to match the redox potentials of cobalt and organic backbone in the corresponding metallopolymers, which has not been explored before.

**Scheme 1:**
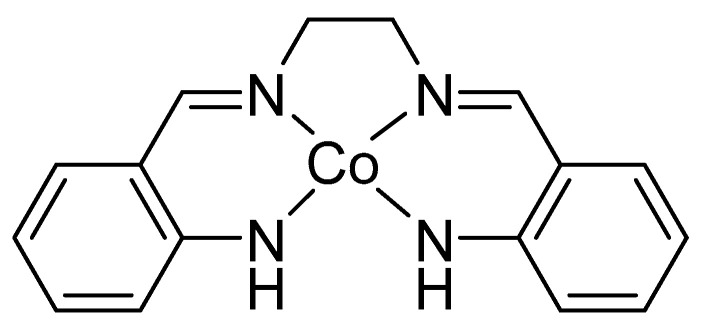
Molecular structure of [Co(Amben)].

“Anilinosalen” N_4_ analogues of N_2_O_2_ [Co(Salen)]-type complexes, also referred to as cobalt(II) complexes of “Amben”-type ligands (Scheme 1), have been studied since the 1970s [21,22,23,24]. Recent research on the electronic structure of one-electron, oxidized, protected [Co(Amben)]-type complexes [25,26] clearly shows that their first oxidation is a ligand-based process yielding Co(II)-anilinyl radical cation species, in contrast to the metal-based first oxidation of [Co(Salen)]-type complexes [3] and corresponding metallopolymers [8,9]. Unprotected [Ni(Amben)] and [Pd(Amben)] exhibiting ligand-based oxidation were demonstrated to undergo electro-oxidative polymerization, resulting in the formation of electroactive polymers [27], and we very recently reported the results of detailed studies on poly-[Ni(Amben)] electrochemistry [28]. [Co(Amben)] is also expected to be prone to electropolymerization. Taking into account the high accessibility of ligand-based redox reactions in [Co(Amben)]-type complexes [25,26], the redox potential of the organic backbone of the corresponding poly-[Co(Amben)] film is expected to be sufficiently close to that of the Co(II)/Co(III) redox couple, thus creating enhanced electroactivity of cobalt centers in the metallopolymer networks. To the best of our knowledge, there have been no reports in the literature on the polymerization of [Co(Amben)] or any similar N_4_ Schiff base cobalt complexes.

In this contribution, we report the successful oxidative electropolymerization of [Co(Amben)] complex. We study the electro-oxidative doping mechanism of a novel poly-[Co(Amben)] polymer by cyclic voltammetry, in situ UV-vis-NIR, EQCM, and electrochemical conductance methods. Characterization of electrochemically induced transformations in conjugated and redox polymers by in situ UV-vis-NIR spectroelectrochemistry is the method of choice for primary identification of electrochemically induced charge carriers in redox active polymers [29]. The EQCM method allows to trace ion and solvent dynamics driven by redox switching in the polymer film during electrochemical charging and discharging [30]. Changes of conductivity with electrochemical doping of the polymer probed by the in situ electrochemical conductance method [31] allows to independently recognize the presence and mobility of different charged states. We use the combined results obtained by in situ electrochemical and spectroelectrochemical techniques to gain insight into the extent of redox matching of the organic backbone and metal centers in poly-[Co(Amben)] films.

## 2. Materials and Methods

### 2.1. Chemicals and Synthesis

Procedures previously described in the literature were used to synthesize the starting ligand H_2_Amben and [Co(Amben)] complex [21]. Their structures were confirmed by CHN elemental analysis for both compounds and ^1^H and ^13^C NMR for the ligand. Ethylene carbonate (Acros Organics, NJ, USA, 99+%) and diethyl carbonate (Sigma-Aldrich, MO, USA 99%) were used as-received. Tetraethylammonium tetrafluoroborate Et_4_NBF_4_ (Sigma-Aldrich, MO, USA 99%) was recrystallized from isopropyl alcohol and dried at 65 °C for 72 h before use. All electrochemical experiments were performed in an argon-filled glove box.

### 2.2. Cyclic Voltammetry and EQCM Studies

Electrochemical measurements were performed with a VSP potentiostat (BioLogic Science Instruments, Seyssinet-Pariset, France). A customized single-compartment three-electrode electrochemical cell was equipped with a glassy carbon plate as the counter electrode and a non-aqueous Ag/Ag^+^ reference electrode (MW‒1085, BASi, IN, USA) filled with a 0.005 mol L^−1^ AgNO_3_ solution in 0.1 M Et_4_NBF_4_/CH_3_CN (its potential was −0.3 V vs. an Ag/AgCl/sat’d NaCl). The numerical values of all potentials reported in this contribution are referred to this reference electrode.

Cyclic voltammetry (CV) experiments were conducted using a platinum disk (BASi, electrode area 0.02 cm^2^) as the working electrode. The cell was filled with a solution containing 0.1 M Et_4_NBF_4_ and 0.001 mol L^−1^ [Co(Amben)] in a 50:50 (*v*/*v*) mixture of ethylene carbonate (EC) and diethyl carbonate (DEC). Cyclic voltammograms were recorded between −0.9 and +1.3 V at a scan rate of 0.1 V s^−1^.

For combined CV/EQCM studies, the VSP potentiostat was synchronized with a 5 MHz QCM100 Quartz Crystal Microbalance equipped with a Metex MXC 1600 frequency counter (Metex Co, Seoul, Korea). A platinum-coated quartz crystal (electrode area 1.37 cm^2^) was used as the working electrode. The oscillation frequency of the crystal was measured by QCM in argon before the immersion in the solution for the polymer deposition. After that, a polymer film was deposited onto the working electrode by potential cycling at a scan rate of 0.05 V s^−1^. Before being subjected to further characterization tests, the polymer-modified crystal was dried in argon until reaching a constant oscillation frequency (for about 30 min). The mass of the dry polymer film was then calculated by using the Sauerbrey equation [32], which relates the mass change per unit area at the electrode surface to the observed change in oscillation frequency of the crystal.

### 2.3. In Situ Conductance Measurements

In situ conductance measurements were carried out in a bipotentiostat regime with the same electrochemical cell as was used for cyclic voltammetry. Interdigitated Pt electrodes (MicruX Technologies, Gijón, Spain) with the comb distance of 5 µm served as the working electrodes. The polymer films were deposited in a CV mode at a scan rate of 0.01 V s^−1^. A constant bias of 5 mV was applied between the combs of the interdigitated electrode and the flowing current was measured as a function of potential. The conductance of the polymer was calculated from the measured current between the combs by applying Ohm’s law [31]. The polymer deposition process was terminated when the conductance reached a plateau (8 cycles), which indicated that the polymer completely filled the gap. The electrodes were then transferred to a monomer-free solution, wherein in situ conductance measurements of the polymer films at 0, 5, 10, and 20 mV biases were performed in parallel with the voltammetric measurements at 0.01 V s^−1^. Linear dependence of conductance maximum vs. applied bias was found.

### 2.4. Scanning Electron Microscopy Measurements

Scanning electron microscopy (SEM) images were obtained using a JEOL JSM 7001 F scanning electron microscope (JEOL Ltd., Tokyo, Japan). The polymer layer grown on the IDE electrode for in situ conductance measurements was analyzed.

### 2.5. UV-vis-NIR Spectroscopy and In Situ Spectroelectrochemical Studies

UV-vis-NIR absorption spectra were collected in 10 mm path-length quartz cuvettes using an SF‒2000 spectrophotometer (OKB Spectr, Saint Petersburg, Russia). The spectrophotometer was combined with a VSP potentiostat for in situ UV-vis-NIR spectroelectrochemical investigation of the polymer films. The working electrode was an indium tin oxide (ITO)-coated conducting glass (Sigma Aldrich, electrode area 1.0 cm^2^). A Pt wire was used as the counter electrode and an AgCl-coated Ag wire was used as the pseudo-reference electrode (its potential was close to that of an Ag/AgCl/sat’d NaCl). Electrodes were placed in a custom-made three-electrode quartz cell. Spectra were acquired in the range 300–1000 nm at fixed potentials, incrementally stepped in 0.1 V intervals from −0.9 to +1.0 V and in 0.15 V intervals from +1.0 to +1.3 V. All spectra were collected under stationary conditions (after the current remained unchanged for 120 s).

## 3. Results

### 3.1. Oxidative Electrochemistry of [Co(Amben)]

**Figure 1:**
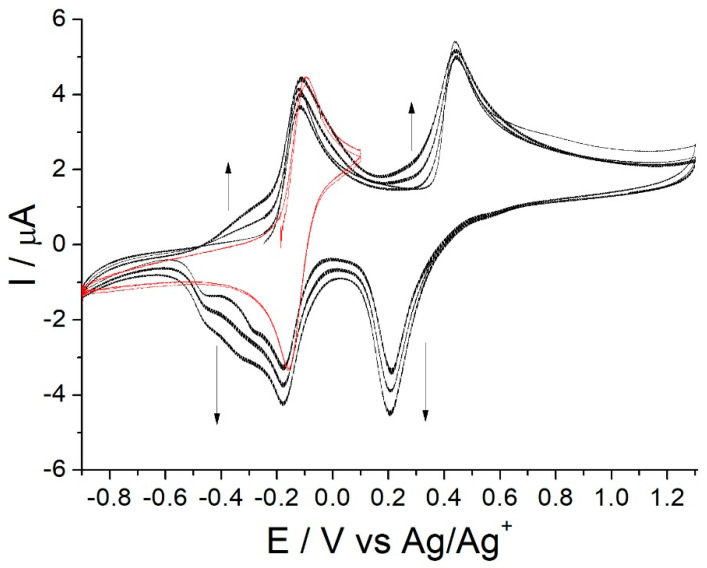
Three consecutive CV cycles of 0.001 mol L^−1^ [Co(Amben)] in 0.1 M Et_4_NBF_4_/EC/DEC between −0.9 and +0.1 V (red curve) or between −0.9 and +1.3 V (black curve) recorded using a Pt disk electrode (0.02 cm^2^) at a scan rate of 0.1 V s^−1^.

The redox properties of [Co(Amben)] were studied in EC/DEC solvent (+0.1 M Et_4_NBF_4_). The cyclic voltammogram of the complex displays two oxidation waves in the potential range from −0.9 to +1.3 V (Figure 1, black curve). The first redox event (E_p/2_ = −0.145 V) appears to be an electrochemically reversible one-electron oxidation of [Co(Amben)] (ΔE_P_ = 55 mV, I_ox_ = I_red_) (Figure 1, red curve). The second oxidation (E_p/2_ = +0.435 V) is accompanied by a reduction wave with peak-to-peak separation of 220 mV, which points to a chemical step following the transfer of the second electron. Potential cycling beyond the second oxidation process leads to the formation of an electroactive film on electrode surface, which could be seen from the appearance of new voltammetric peaks and an increase in peak currents after the first CV cycle in the potential range between −0.9 and +1.3 V, as indicated by arrows in Figure 1.

### 3.2. EQCM Study of the Electro-Oxidative Polymerization of [Co(Amben)]

**Figure 2:**
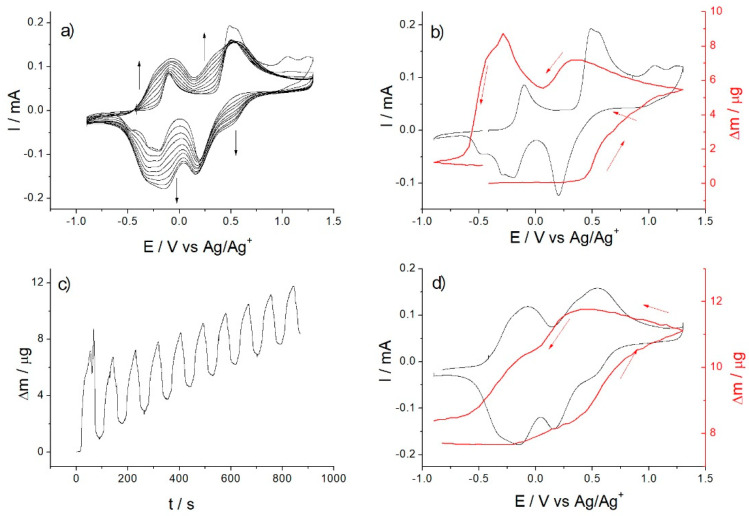
EQCM-monitored polymerization of 0.001 mol L^−1^ [Co(Amben)] in 0.1 M Et_4_NBF_4_/EC/DEC performed by 10 CV scans in the potential range between −0.9 and +1.3 V at a scan rate of 0.05 V s^−1^: (**a**) the current-potential response, (**b**) the current-potential response (black curve) and the corresponding electrode mass variation vs. potential (red curve) during the first CV cycle, (**c**) the electrode mass variation vs. time for 10 consecutive CV cycles, (**d**) the current-potential response (black curve) and the corresponding electrode mass variation vs. potential (red curve) during the 10th CV cycle.

We used the EQCM technique to closely monitor the electropolymerization of [Co(Amben)]. The polymer film was deposited onto the EQCM crystal by 10 cycles of electrode polarization in a CV mode (Figure 2a).

In the first CV cycle, no mass change occurs prior to the second oxidation of the monomer (Figure 2b), which agrees well with the electrochemically reversible nature of the first redox process. The onset of the polymer deposition coincides with the onset of the second oxidation of [Co(Amben)]. Mass growth continues until CV currents become negative at +0.35 V in the reverse scan. As the current becomes cathodic, the electrode mass starts to decrease, which could be associated with egress of charge-compensating ions during de-doping of the deposited polymer film. The broad reduction wave observed between +0.05 and −0.6 V is accompanied by a mass increase in the potential range from +0.05 to −0.28 V, followed by a sharp decrease in the electrode mass between −0.28 and −0.6 V. The overall mass vs. potential dependence in the first cycle of electrochemical polymerization is quite complex and appears to be a manifestation of at least two processes: the electro-oxidative polymerization accompanied by electrochemical doping and de-doping of the emerging polymer film and the deposition/dissolution of oligomers at the end of the first CV scan. The relative contribution of the first process to the registered mass changes increases and the contribution of the oligomers’ deposition/dissolution decreases as the deposited polymer mass increases in the following polymerization CV cycles (Figure 2c,d).

During the polymer deposition, the total gain in the electrode mass was 1.06 µg at the end of the first cycle and 8.39 µg after 10 cycles, demonstrating reasonable linear dependence of the polymer mass on the number of CV cycles (Figure 2c).

**Scheme 2:**
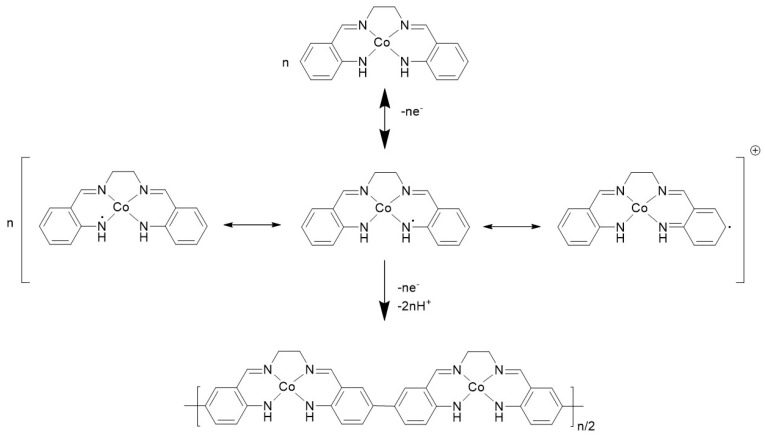
Mechanism of [Co(Amben)] electro-oxidative polymerization.

Based on these findings combined with the data on the electronic structure of the analogous protected [Co(Amben)]-type complex [25,26], we propose the following polymerization mechanism of [Co(Amben)], as in Scheme 2.

### 3.3. CV and EQCM Studies of Thin Poly-[Co(Amben)] Films

**Figure 3:**
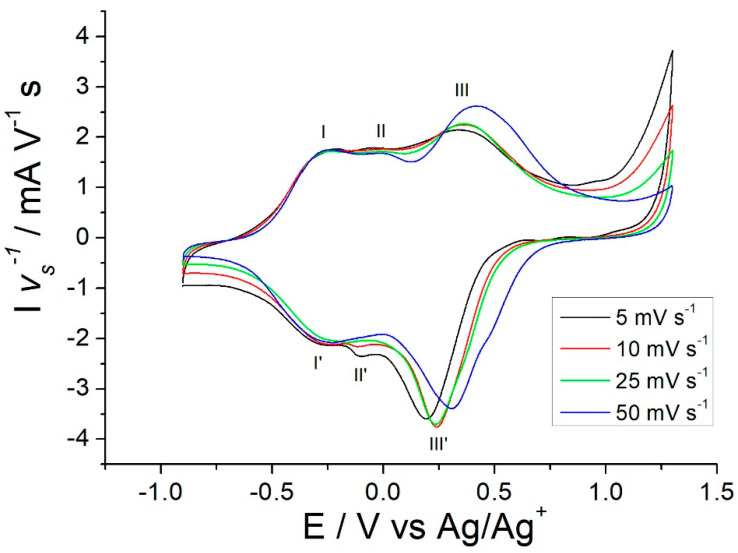
Scan rate-normalized cyclic voltammograms of poly-[Co(Amben)] in 0.1 M Et_4_NBF_4_/EC/DEC in the potential range between −0.9 and +1.3 V at different scan rates.

Poly-[Co(Amben)]-modified EQCM electrode was transferred to the monomer-free electrolyte solution and completely de-doped by applying the potential of −0.9 V for 2 min. After that, it was washed with pure solvent and dried. The mass of the dry de-doped film was measured to be 7.0 µg. The polymer-modified electrode was immersed in the monomer-free electrolyte solution and CV curves were obtained at 50, 25, 10, and 5 mV s^−1^ sweep rates (Figure 3), with simultaneous oscillation frequency registration.

At sweep rates of 5–25 mV s^−1^, the anodic and cathodic peak currents display linear dependence on the sweep rate, which can be seen from almost equal peak currents in the scan rate-normalized CV representation in Figure 3. It confirms that the film operates in a thin-layer regime. The charge that reversibly cycled through the film at these sweep rates also remained almost constant.

Three pairs of oxidation/reduction peaks are observed in the cyclic voltammograms of poly-[Co(Amben)]: −0.24 and −0.27 V (I, I′), 0 and −0.1 V (II, II′), and +0.35 and +0.24 V (III, III′) (values of peak potentials are given for the CV curve recorded at 10 mV s^−1^ scan rate).

The number of electrons exchanged by each [Co(Amben)] unit of the polymer film in the redox processes, *n*, was calculated using Equation (1) [33]:

(1) $$n=\frac{Q_{CV}\cdot M}{F\cdot m_{QCM}}$$

where *Q_CV_* is the voltammetric reduction charge, in C, *M* is the molar mass of repeat unit in the undoped polymer, in g mol^−1^, *F* is the Faraday constant (96,485 C mol^−1^), and *m_QCM_* is the mass of the dry undoped polymer film determined by microgravimetric measurements, in g.

The calculated value of *n* = 1.25 corresponds to the reversible transfer of a maximum of 1.25 electrons per repeat unit of poly-[Co(Amben)] film during its electrochemical doping/de-doping.

The specific capacity of poly-[Co(Amben)], *C_mAh_*, can be calculated using the following equation:

(2) $$C_{mAh}=\frac{n\cdot F}{3.6\cdot M}$$

The average specific capacity of poly-[Co(Amben)] calculated by substituting the value of *n* = 1.25 into Equation (2) is 104 mAh g^−1^.

**Figure 4:**
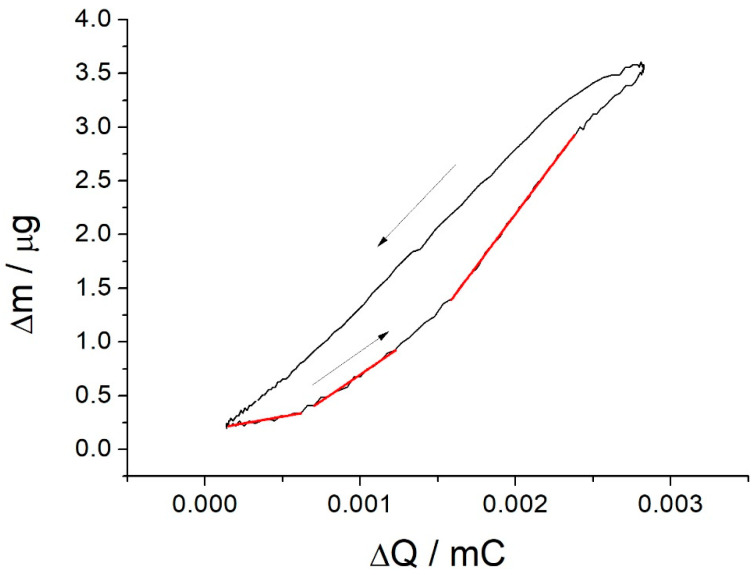
Mass-charge plot (Δm vs. ΔQ) for the CV/EQCM test of the poly-[Co(Amben)] film conducted at a scan rate of 25 mV s^−1^ (black curve) and linear regions used to calculate the molar masses of injected mobile charge carriers (red lines).

Electrochemical charge injection into a redox active polymer is accompanied by ion transfer into or out of the film to maintain its electroneutrality. The EQCM technique provides information on the effective molar mass of charge carriers. The Δm vs. ΔQ curve of the anodic part of the CV cycle of poly-[Co(Amben)] clearly displays three different linear regions corresponding to different molar masses of exchanged electrolyte species (Figure 4). Calculated effective molar masses of charge carriers and potential intervals of the linear regions are presented in Table 1**.**

### 3.4. In Situ UV-vis-NIR Spectroelectrochemical Study of Poly-[Co(Amben)] Oxidation

Spectroelectrochemical characterization of redox transformations in electrochemically active polymers is a powerful tool to unveil the nature of electrochemically induced charge carriers in the doped polymer films. For spectroelectrochemical studies, the polymer was deposited on ITO-coated glass by 30 CV cycles at 50 mV s^−1^ in the potential range from −0.9 to +1.3 V. After the polymer deposition, the polymer-modified ITO electrode was washed with pure solvent and transferred to the spectroelectrochemical cell.

**Figure 5:**
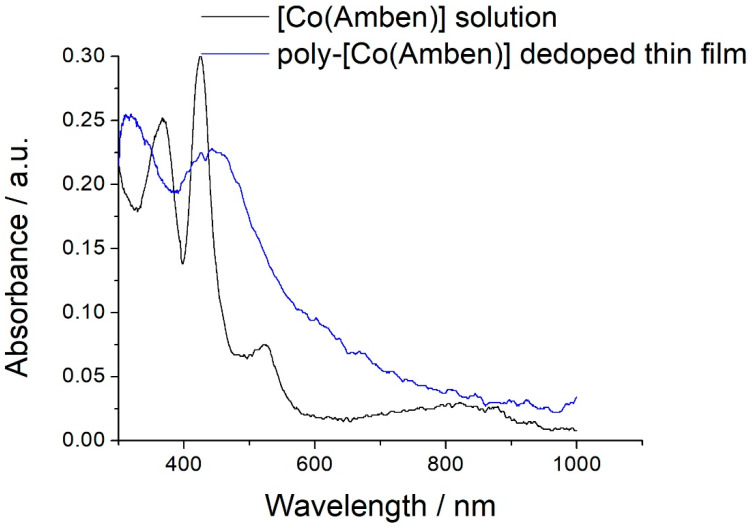
UV-vis-NIR spectra of 0.33*10^−4^ M [Co(Amben)] solution (black curve) and poly-[Co(Amben)] polymer film in the neutral (de-doped) state (blue curve).

The low-intensity NIR band in the UV-vis-NIR solution spectrum of [Co(Amben)] (Figure 5, black curve) could be assigned to the d-d transition. The adsorption maxima at 522 and 425 nm originate from MLCT transitions. The absorption band with a maximum at 356 nm could be described as the LLCT band [21,22,23,24,25,26]. The optical spectrum of the de-doped poly-[Co(Amben)] film Figure 5, blue curve) resembles that of the monomer but is red-shifted and features broadened adsorption maxima.

**Figure 6:**
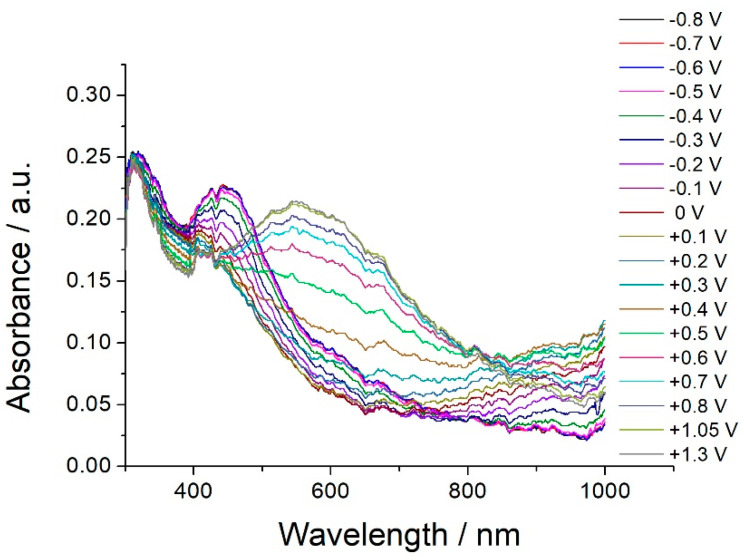
UV-Vis-NIR spectra of a poly-[Co(Amben)]-modified ITO-coated glass electrode acquired during redox switching in 0.1 M Et_4_NBF_4_/EC/DEC.

UV-vis-NIR spectra of the doped poly-[Co(Amben)] film were collected at fixed potentials, incrementally stepped in 0.1 V intervals from −0.9 to +1.0 V and in 0.15 V intervals from +1.0 to +1.3 V (Figure 6).

**Figure 7:**
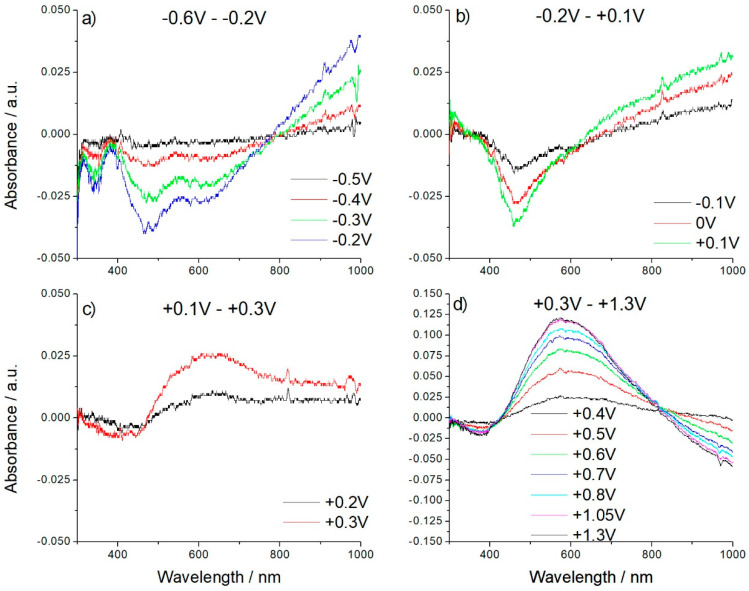
UV-vis-NIR spectra of a poly-[Co(Amben)]-modified ITO-coated glass electrode acquired during redox switching in 0.1 M Et_4_NBF_4_/EC/DEC: (**a**) spectra collected from −0.6 to −0.2 V, referenced to the spectrum of the polymer at −0.6 V, (**b**) spectra collected from −0.2 to +0.1 V, referenced to the spectrum of the polymer at −0.2 V, (**c**) spectra collected from +0.1 to +0.3 V, referenced to the spectrum of the polymer at +0.1 V, (**d**) spectra collected from +0.3 to +1.3 V, referenced to the spectrum of the polymer at +0.3 V.

Four sets of isosbestic points were observed during electrochemical oxidation of the polymer. Spectral changes at different electrochemical conversion stages can be better assessed using differential absorbance spectra referenced to the spectra obtained in the beginning of each stage (Figure 7).

Initial oxidation up to the potential of −0.2 V results in a decrease in all high-energy absorption and the development of a broad NIR absorption with a maximum above 1000 nm (Figure 7a). These changes are accompanied by an isosbestic point at ca. 780 nm. We attribute them to the oxidation of benzidine moieties resulting in the formation of benzidine radical cations in the polymer structure [25,34,35,36].

The second stage of the film oxidative transformation occurs between −0.2 and +0.1 V and is accompanied by isosbestic points at ca. 350 and 670 nm (Figure 7b). The broad NIR absorption continues to increase monotonically, as in the first stage. The band at 300 nm also increases in intensity. The only band depleted at this stage is the one at ca. 460 nm, which could be assigned to an MLCT band. Similar potential-induced UV-vis-NIR spectral changes were observed during the first oxidation of isoelectronic [Co(Salen)]-type monomeric [37] and polymeric [9] complexes, and were assigned to the Co(II)/Co(III) conversion. The NIR absorbance has been shown to be a spectroscopic marker for a ligand contribution to the electronic structure of the cations [37]. Thus, the second oxidation stage of poly-[Co(Amben)] could be assigned to the Co(II)/Co(III) conversion.

The third spectroscopically identified oxidation stage is observed in the potential range from +0.1 to +0.3 V and accompanied by a new isosbestic point at ca. 450 nm (Figure 7c). The broad NIR absorbance continues to increase in intensity, the absorbance below 450 nm depletes, and the new intense absorption band at ca. 600 nm appears and grows. We assign the NIR absorbance growth and the high-energy bands depletion to a continuing conversion of neutral fragments to benzidine radical cations with Co(III) contribution. The new intense absorption band developing at 600 nm could be assigned to the formation of benzidine di-cations in the film structure [34,36,37].

The fourth stage of the polymer film oxidation is observed at potentials above +0.4 V and accompanied by isosbestic points at 425 and 825 nm (Figure 7d). The depletion of the NIR band and the band at 400 nm and further development of the intense band at 600 nm are observed. We propose that the conversion of benzidine radical cations to di-cations is the reason for the observed spectral changes.

### 3.5. In Situ Conductance and SEM Studies of Thick Poly-[Co(Amben)] Films

**Figure 8:**
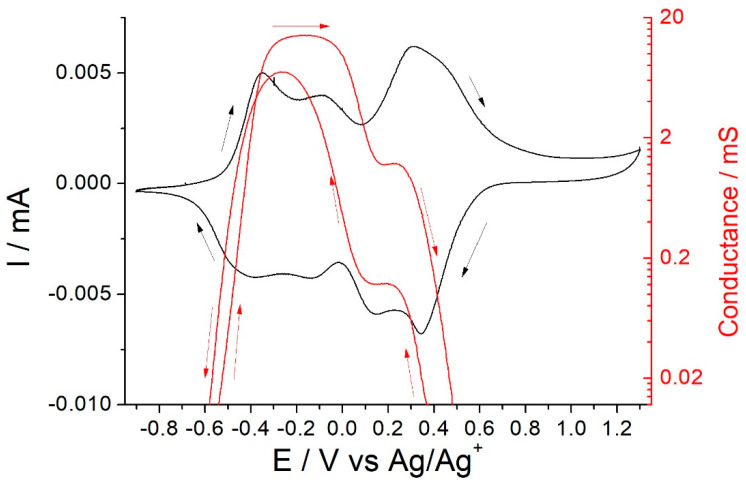
Cyclic voltammogram (black curve) and corresponding conductance profile (red curve) of a poly-[Co(Amben)]-coated interdigitated electrode in 0.1 M Et_4_NBF_4_/EC/DEC at a scan rate of 10 mV s^−1^ with 5 mV bias.

In situ electrochemical conductance measurements were used to investigate the dependence of poly-[Co(Amben)] conductivity on the electrode potential (and hence, the doping level of the metallopolymer) and gain insight into the nature of electrochemically induced charge carriers in the polymer film. The CV curve of poly-[Co(Amben)] deposited between the combs of an interdigitated electrode (IDE) and the simultaneously obtained conductance profile are presented in Figure 8.

The conductivity of the de-doped polymer film (at potentials below −0.55 V) is negligible. Electrochemical oxidation induces the formation of mobile charge carriers in the polymer, and the conductance sharply increases from the onset of oxidation until it reaches the first plateau of maximum conductivity in the range of potentials between −0.3 and 0 V. With further oxidation, the film conductivity decreases, however the second plateau of conductivity is observed between +0.15 and +0.3 V. After that, the conductivity of the film continues to decrease until it becomes negligible at potentials more positive than +0.5 V. The conductance vs. potential curve in the reverse scan resembles that in the forward scan with a small hysteresis, demonstrating that conductivity changes with the film doping level are reversible.

**Figure 9:**
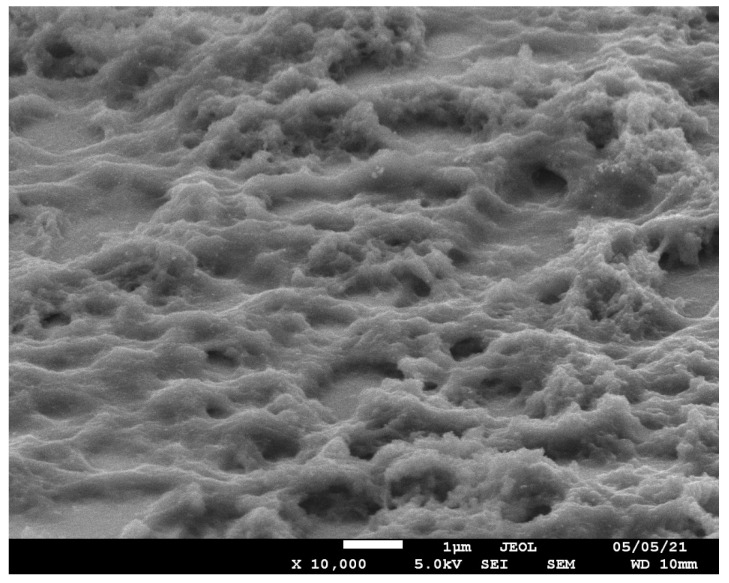
SEM image of poly-[Co(Amben)]-coated interdigitated electrode.

The SEM image of the poly-[Co(Amben)]-coated IDE electrode (Figure 9) reveals that the polymer film continuously and fully covers both the Pt-coated bands and the glass inter-bands’ space. The polymer displays non-uniform morphology. It consists of a thin dense bottom layer and a porous top layer featuring fibrous and globular domains.

## 4. Discussion

**Scheme 3:**
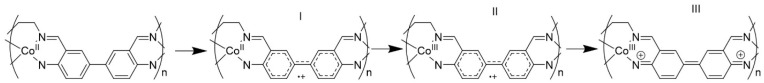
Mechanism of poly-[Co(Amben)] electrochemical doping.

We used several in situ electrochemical and spectroelectrochemical methods to characterize redox transitions in poly-[Co(Amben)], and all of them clearly show three different electrochemically induced redox states in the doped polymer films. Here, we combine all these findings and discuss the redox transformations in poly-[Co(Amben)] films. As CV, EQCM, in situ UV-vis-NIR, and conductance measurements were performed on the polymer films with different thicknesses, the exact potential ranges for the existence and concentration of different charge carriers as well as the number of redox peaks and their potentials in CV curves could reasonably vary for different methods. Good agreement between the electrochemical data obtained for thin (Figure 3) and thick (Figure 8) poly-[Co(Amben)] films indicates that the types of charge carriers appear to be virtually independent of the film thickness. As all the electrochemically induced transformations in the polymer film are reversible, below we discuss the transformation of the neutral (de-doped) film to the fully oxidized state (the spectroscopically identified potential ranges for different oxidation stages are presented) (Scheme 3).

Stage I, from −0.5 to −0.2 V: radical cation formation.

The onset for poly-[Co(Amben)] oxidation is located at −0.55 V. Electrochemical oxidation induces the formation of radical cation species in the film, as follows from the analysis of UV-vis-NIR optical transitions and the rapid increase in the film conductivity. This redox process is accompanied by the transfer of electrolyte species with equivalent mass of 25 g mol^−1^, as identified by EQCM. That formally equals the inclusion of one BF_4_^−^ anion (86.8 g mol^−1^), accompanied by the expulsion of half of a DEC solvent molecule (118.1 g mol^−1^) per one electron exchanged.

Stage II, from −0.2 to +0.1 V: Co(II)/Co(III) conversion.

The existence of metal-centered oxidation of poly-[CoAmben)] in this potential range follows from the analysis of UV-vis-NIR spectral changes. The EQCM-monitored transfer of electrolyte species at this stage is consistent with ingress of one BF_4_^−^ anion into the film per one electron exchanged. This potential range for Stage II coincides with the range of potentials for constant maximum conductivity values. Plateau-like conductivity between −0.3 and 0 V could be attributed to the superposition of two mixed-valence conductivity regimes [1]. The first one can be assigned to the generation of a benzidine radical cation/neutral system, which manifests itself as peak I in the CV curve of the polymer (Figure 3) and is expected to result in the conductivity maximum at −0.24 V. The second mixed-valence conductivity regime can be ascribed to the generation of Co(III)/Co(II) states, which manifests itself as peak II in the CV curve of the polymer (Figure 3) and is expected to result in the conductivity maximum at 0 V. An increase in the concentration of radical cations with an increase in the electrode potential causes a sharp drop in the polymer film conductivity in the range of potentials from 0 to +0.15 V.

Stage III, from +0.1 to +0.75 V: di-cation formation.

The appearance of the absorption band at 600 nm in the optical spectra of poly-[Co(Amben)] at potentials above +0.1 V indicates the formation of benzidine di-cations in the film structure. In the potential range from + 0.1 to +0.3 V, this process is still accompanied by the oxidation of remaining neutral repeat units of the polymer into benzidine radical cations, as follows from the continuing increase in the NIR absorbance. At potentials above +0.3 V, the only spectroscopically identified process is the conversion of benzidine radical cations to di-cations. This electrochemical conversion is accompanied by the incorporation of electrolyte species with molar mass of 185 g mol^−1^ per one electron exchanged in the redox process at the potentials above +0.35 V, as monitored by EQCM. This mass exchange is numerically consistent with the entrance of one BF_4_^−^ anion and one molecule of EC (88.06 g mol^−1^) per one electron extracted from the polymer film. The local conductivity maximum is observed at +0.3 V (Figure 8), which is close to the peak III potential and could thus be attributed to the mixed valence conductivity between benzidine di-cation/radical cation states.

In contrast to polymeric cobalt complexes of N_2_O_2_ Salen-type ligands exhibiting the Co(II)/Co(III) conversion at lower potentials than the potentials of organic backbone oxidation [8,9], the findings of this study demonstrate that the metal-centered redox activity in poly-[Co(Amben)] is located at potentials matching the redox potentials of the organic backbone (more specifically, between the potentials of neutral/radical cation and radical cation/di-cation redox conversions) and corresponding to a highly conductive state of the polymer film. Such difference is apparently the effect of replacing phenoxo oxygen by anilido nitrogen in the coordination sphere of the monomer. Unfortunately, the conductivity profiles of poly-[Co(Amben)] and poly-[Co(Salen)] cannot be directly compared because an attempt to deposit the required amount of poly-[Co(Salen)] on the IDE electrode failed and there are no reports in the literature on the successful in situ electrochemical conductance measurements of the polymer films obtained from [Co(Salen)] monomers without heterocyles attached in the *para*-positions of the phenyl rings. We believe that sufficiently thick poly-[Co(Salen)] films cannot be grown because of their low conductivity, which is directly related to the separation of metal- and ligand-based oxidation in terms of potential.

Replacing two oxygen atoms with two nitrogen ones in the cobalt coordination site allowed us to obtain the same effect as was shown previously for EDOT- [10,11] and bithiophene [20]-functionalized [Co(Salen)] derivatives: the matching the Co(II)/Co(III) redox potential to the oxidation potential of the organic backbone is observed in poly-[Co(Amben)]. As a result, this polymer can be electrochemically grown as thick films while retaining efficient electrochemical conversions (metal- and ligand-based) of the thin films.

Three different redox states found to exist in the poly-[Co(Amben)] film could potentially enable the reversible redox transfer of three electrons per a polymer repeat unit, however, the experimentally identified maximum oxidation state of the polymer corresponds to the transfer of only 1.25 electrons per repeat unit of poly-[Co(Amben)] film, which is close to the maximum doping level shown previously for poly-[Co(Salen)] [9]. In the case of poly-[Co(Amben)], the redox matching of the organic backbone and metal centers did not result in the enhancement of its capacitive properties, as compared with its N_2_O_2_ analogue. Further tuning of steric and electronic properties of the Amben-type monomer is expected to be effective in increasing the polymer capacity up to theoretical levels [9,38]. Understanding and overcoming factors that prevent the complete reversible oxidation of the polymeric cobalt complex of N_4_ Amben-type ligand will open up the possibility of using this type of materials in various energy-storage applications. Retaining metal-centered redox activity in thick poly-[Co(Amben)] films should enable the creation of functional materials for sensing and electrocatalytic applications based on cobalt-mediated redox reactions.

## 5. Conclusions

We found that the cobalt(II) complex of bis(*o*-aminobenzylidene)ethylenediamine N_4_ Schiff base, [Co(Amben)], could be electropolymerized in EC/DEC solvent when subjected to the potentials of the second accessible oxidation. The resulting novel polymer, poly-[Co(Amben)], is conductive and electrochemically active and demonstrates three electrochemically accessible redox states, independent of the film thickness. The consecutive formation of three redox states during reversible electrochemical oxidation of the polymer film was confirmed by the combination of cyclic voltammetry, in situ UV-vis-NIR, EQCM, and electrochemical conductance methods. By analyzing the potential-dependent changes in the optical, gravimetric, and conductive properties of the polymer, we assigned these states to benzidine radical cations, Co(III) ions, and benzidine di-cations. We demonstrated that the Co(II)/Co(III) redox conversion in poly-[Co(Amben)] films occurs at potentials that closely match the redox potentials of the organic backbone and correspond to the potentials of the high polymer conductivity. Efficient metal-centered redox reactions present a distinct advantage of the novel polymer over its N_2_O_2_ analogue and make it promising for sensing and electrocatalytic applications.

## Figures and Tables

**Table 1:** Mass of charge-compensating species entering poly-[Co(Amben)] film during oxidation at different potential ranges.

Potential Range	Charge Carrier Molar Mass, g mol^−1^
−0.5 to −0.15 V	25
−0.15 to +0.2 V	94
+0.35 to +0.95 V	185

## Data Availability

The data presented in this study are available on request from the corresponding author.
